# Case Report: Myopathy in Critically Ill COVID-19 Patients: A Consequence of Hyperinflammation?

**DOI:** 10.3389/fneur.2021.625144

**Published:** 2021-01-29

**Authors:** Viviana Versace, Luca Sebastianelli, Davide Ferrazzoli, Leopold Saltuari, Markus Kofler, Wolfgang Löscher, Antonino Uncini

**Affiliations:** ^1^Department of Neurorehabilitation, Hospital of Vipiteno (SABES-ASDAA), Vipiteno, Italy; ^2^Department of Neurology, Hochzirl Hospital, Zirl, Austria; ^3^Department of Neurology, Medical University Innsbruck, Innsbruck, Austria; ^4^Department of Neuroscience, Imaging and Clinical Sciences, University “G. d'Annunzio”, Chieti, Italy

**Keywords:** COVID-19, SARS-CoV-2, critical illness myopathy, compound muscle action potential duration, interleukin 6

## Abstract

**Introduction:** COVID-19-associated muscular complications may comprise myalgia, weakness, wasting, and rhabdomyolysis. Skeletal muscle damage in COVID-19 may be due to direct infection by the virus SARS-CoV-2 through interaction with the ACE2 receptor, systemic hyper-inflammatory state with cytokine release and homeostatic perturbation, an autoimmune process, or myotoxic drugs. Disclosing the cause of weakness in an individual patient is therefore difficult.

**Case Description:** We report two patients, who survived typical COVID-19 pneumonia requiring intensive care treatment and who developed early on myalgia and severe proximal weakness in all four limbs. Laboratory exams revealed elevated serum creatine kinase and markedly increased C-reactive protein and interleukin 6, concurring with a systemic inflammatory response. On admission in neurorehabilitation (4 and 7 weeks after COVID-19 onset, respectively), the patients presented with proximal flaccid tetraparesis and limb-girdle muscle atrophy. Motor nerve conduction studies showed decreased amplitude and prolonged duration of compound muscle action potentials (CMAPs) with normal distal motor latencies and normal conduction velocities in median and ulnar nerves. Needle electromyography in proximal muscles revealed spontaneous activity in one and myopathic changes in both patients.

**Discussion:** Clinical, laboratory, and electrodiagnostic findings in these patients were unequivocally consistent with myopathy. Interestingly, increased distal CMAP duration has been described in patients with critical illness myopathy (CIM) and reflects slow muscle fiber conduction velocity due to membrane hypo-excitability, possibly induced by inflammatory cytokines. By analogy with CIM, the pathogenesis of COVID-19-related myopathy might also depend on hyperinflammation and metabolic pathways that may affect muscles in a pathophysiological continuum from hypo-excitability to necrosis.

## Introduction

Muscular complications in hospitalized coronavirus disease (COVID-19) patients may include myalgia, muscle weakness and wasting, elevated serum creatine kinase (CK), and rhabdomyolysis (1). Severe acute respiratory syndrome coronavirus 2 (SARS-CoV-2) binds to cells through the angiotensin-converting enzyme 2 (ACE2) receptor, which is expressed in skeletal muscle (2). However, SARS-CoV-2 particles, despite its broad organotropism beyond the respiratory tract, have not been demonstrated in muscle samples so far (3).

Besides the possibility of direct skeletal muscle injury by SARS-CoV-2, other conceivable causes of myopathies in COVID-19 may comprise an autoimmune process, such as in necrotizing autoimmune myositis, consequence of the systemic hyperinflammatory state, and myotoxicity by medication (e.g., hydroxychloroquine, anti-retroviral agents) (2, 4, 5). Moreover, severely affected COVID-19 patients with systemic inflammatory response, prolonged intensive care treatment with ventilation, and immobilization are prone to develop critical illness myopathy (CIM). Hence, explaining the exact cause of weakness in an individual patient may be difficult.

To date, CIM has been reported and at least electrodiagnostically confirmed in 20 patients with COVID-19 (6–9) (Table 1). Intensive care unit acquired muscle weakness was clinically diagnosed in 72% of COVID-19 patients at awakening (11). Compared to patients without muscle weakness, myopathic patients had longer ICU stays, prolonged duration of invasive mechanical ventilation, higher mean morning glycemia, higher exposure to corticosteroids, sedatives, analgesics, and neuromuscular blocking agents (11). Half of critically ill COVID-19 patients presented acute myopathy in a recent retrospective study (10).

**Table 1:** Studies on COVID-19-patients with intensive care unit acquired myopathy.

	**N of patients**	**Age mean (range) [years]**	**Sex**	**ICU stay mean (range) [days]**	**Medication**	**Clinical feature**	**NCS/EMG**	**Muscle biopsy**	**CK peak-level**	**IL-6 peak-level**
(9)	7	NA	NA	NA	Antiretrovirals, neuromuscular blockers, corticosteroids, antibiotics	Generalized muscular weakness	Myopathy	Three patients (scattered necrotic and regenerative fibers, no inflammatory infiltrates)	181–3,228 μmol/l	N/A
(10)	5	N/A	N/A	N/A	Antirheumatics, antiretrovirals, corticosteroids, antibiotics	Generalized muscular weakness	Myopathy	ND	61–1,206 μg/l	NA
(8)	6	61 (51-72)	1 F	6-14 until NCS/EMG	Antirheumatics, antiretrovirals, corticosteroids, antibiotics, anticoagulants	Acute flaccid quadriplegia	Myopathy; reduced CMAP amplitude with markedly prolonged duration	ND	55–1,274 UI/L	18.4–5,402.2 ng/ml
(6)	1	68	M	65	Antibiotics	Severe symmetrical proximal and distal weakness and diffuse muscle wasting	Myopathy and bilateral peroneal compression neuropathy.	ND	NA	NA
(7)	1	62	F	30	Antirheumatics, antiretrovirals, antibiotics, neuromuscular blockers, antifungal drugs, corticosteroids.	Symmetrical muscle weakness predominant in lower limbs and proximal muscles.	Myopathy	ND	Normal	NA

*ICU, intensive care unit; NCS/EMG, nerve conduction studies/electromyography; CK, creatine kinase; IL-6, interleukine 6; F, female; M, male; CMAP, compound muscle action potential; ND not done; NA not available*.

## Case Description

Here, we report two patients who survived typical COVID-19 pneumonia, confirmed by RT-PCR test on nasopharyngeal swab and by chest computed tomography, which showed bilateral diffuse consolidations and ground-glass opacities. No personal or family medical history of rhabdomyolisis or myoglobinuria or any type of muscle pathology was known. Neither patient had ever received statin therapy or other potentially myotoxic agents. In general, the patients did not suffer from any previous relevant pathology.

Table 2 summarizes demographic, clinical, laboratory, and electrophysiological data.

**Table 2:** Demographic, clinical, laboratory, and electrodiagnostic data.

**Patient**	**Age**	**Sex**	**ICU stay**	**Clinical features**	**Laboratory findings (peak levels)**						**NCS/EMG: time since disease onset**	**Sensory NCS**		**Motor NCS**				**EMG**
					**CK**	**CRP**	**IL-6**	**D-dimer**	**WBC**	**Lymphocyte**		**SNAP amplitude**	**sNCV**	**CMAP amplitude**	**CMAP duration**	**DML**	**mNCV**	Proximal muscles^*^
			**[weeks]**		**[U/l]**	**[mg/l]**	**[pg/ml]**	**[mg/l]**	**[×10^**3**^/μl]**	**[×10^**3**^/μl]**	**[weeks]**	**[μV]**	**[m/s]**	**[mV]**	**[ms]**	**[ms]**	**[m/s]**	
					***40–220***	***<0.8***	***<7.0***	***<0.5***	***3.6–10.5***	***1.1–4.5***								
1	77	M	6	Proximal weakness and muscle wasting in upper more than lower limbs; myalgia; fatigue	4,002	15.9	225.2	1.5	10.1	2.8	7	L median: 15.2	50	L median: 4.2	**9.3**	3.9	48	Myopathic
												R ulnar: 12.4	48	R ulnar: 5.7	**13.7**	2.9	55	
												R sural: 6.3	47	L peroneal: 2.1	7.3	3.1	40	
														R tibial: 2.4	4.2	4.0	41	
2	58	M	3	Predominantly proximal weakness in four limbs; hyporeflexia; myalgia	6,732	17.1	343.6	2.1	9.9	4.6	4	L median: 22.5	49	L median: 3.9	**9.0**	3.8	48	Myopathic
												R ulnar: 25.8	51	R ulnar: 4.8	**8.7**	2.5	50	
												R sural: 9.5	48	L peroneal: 1.2	6.5	4.0	39	
														R tibial: 2.2	6.7	4.1	40	

*Laboratory data were obtained during intensive care. Clinical and electrodiagnostic data were obtained during neurorehabilitation*.

*M, male; F, female; ICU, intensive care unit; CK, creatine kinase; CRP, C-reactive protein; WBC, white blood cell count; IL, interleukin; NCS, nerve conduction study; EMG, electromyography; SNAP, sensory nerve action potential; sNCV, sensory nerve conduction velocity; CMAP, compound muscle action potential; DML, distal motor latency; mNCV, motor nerve conduction velocity; R, right; L, left*.

*The upper limits of normality (mean + 2 SD) for distal CMAP duration using a low frequency filter of 2 Hz are: median nerve = 7.3 ms, ulnar nerve = 7.5 ms, peroneal nerve = 7.3 ms, tibial nerve = 6.8 ms (12); abnormal values are marked in bold*.

* *Left deltoid, right triceps brachii, left iliopsoas, and right rectus femoris muscles*.

Because of respiratory failure, both patients required intensive care treatment, including tracheostomy and ventilatory support for several weeks. Oral treatment with hydroxychloroquine 200 mg twice a day and lopinavir/ritonavir 400/100 mg twice a day was administered for 3 weeks. No antibiotics, corticosteroids or analgesics were administered. High serum levels of C-reactive protein (CRP) and interleukin 6 (IL-6) were documented during the acute phase (Table 2).

After weaning from sedation (intravenous sufentanil/propofol together with rocuronium bromide as muscle relaxant) and ventilation, the patients suffered mild dyspnea requiring oxygen support (2 l/min), complained of myalgia and fatigue, and showed on examination severe proximal muscles weakness in in both upper and lower limbs (Medical Research Council scale 2/5). Strength in distal muscle was normal. Deep tendon reflexes were hypoactive. No deep or superficial sensory disturbance was noted. Cranial nerve examination was unremarkable; in particular, no bulbar muscles weakness was found.

Laboratory examination revealed elevated creatine kinase (CK) (peak-levels 4,002 and 6,732 U/l, respectively) which progressively normalized in the following 3 weeks, but no myoglobinuria nor acute renal failure signs. Due to the Covid-19-related emergency situation in Italian Intensive Care Units, no further neuroradiological or histopathological muscle studies could be performed.

**Figure 1:**
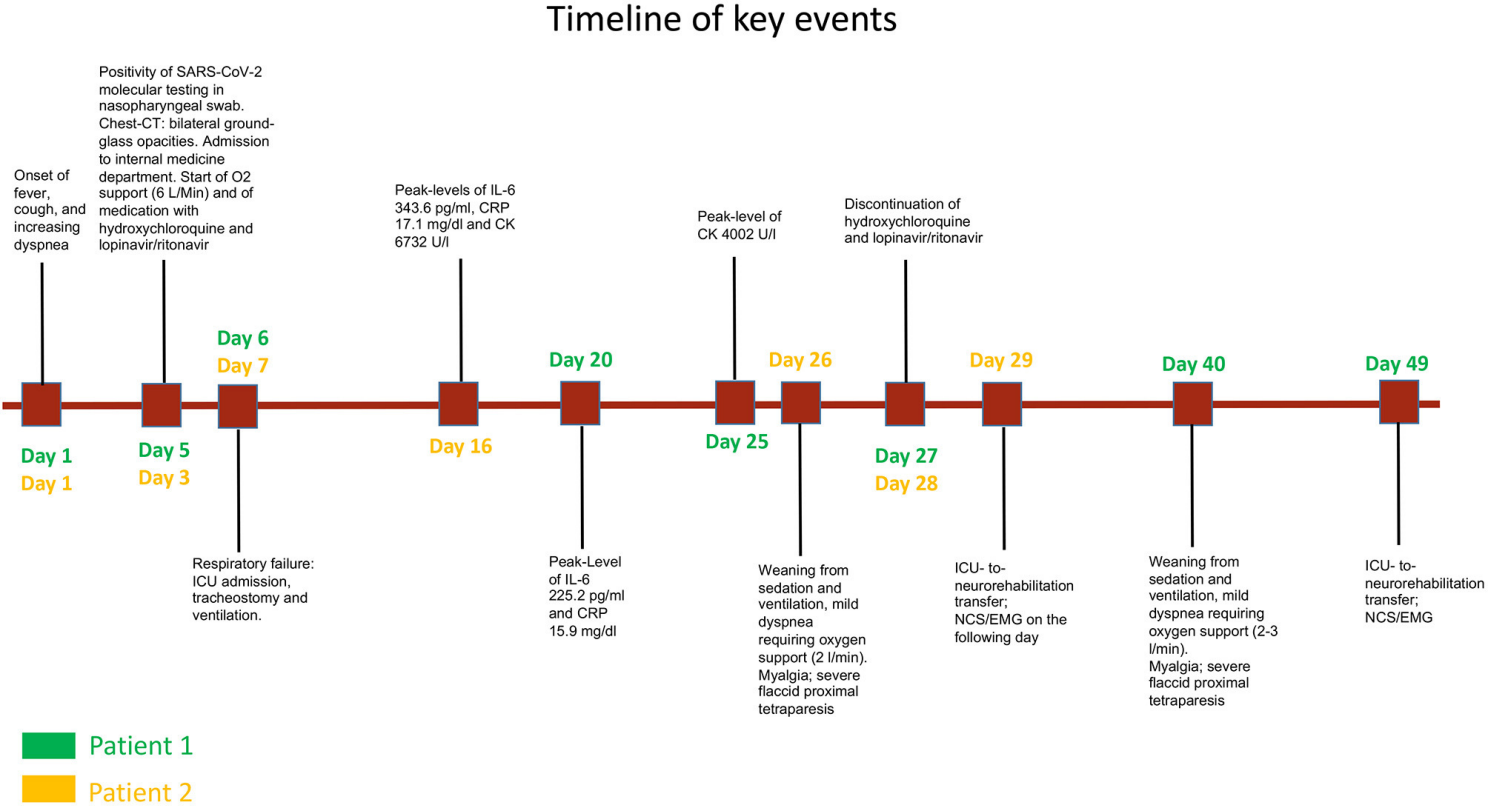
Timeline of key events related to COVID-19 and myopathy in patient 1 (green labels) and patient 2 (yellow labels).

On admission in the neurorehabilitation unit (4 and 7 weeks after onset of COVID-19, respectively), both patients presented with flaccid proximal tetraparesis and limb-girdle muscle atrophy. A timeline of the clinical course is presented in Figure 1.

**Figure 2:**
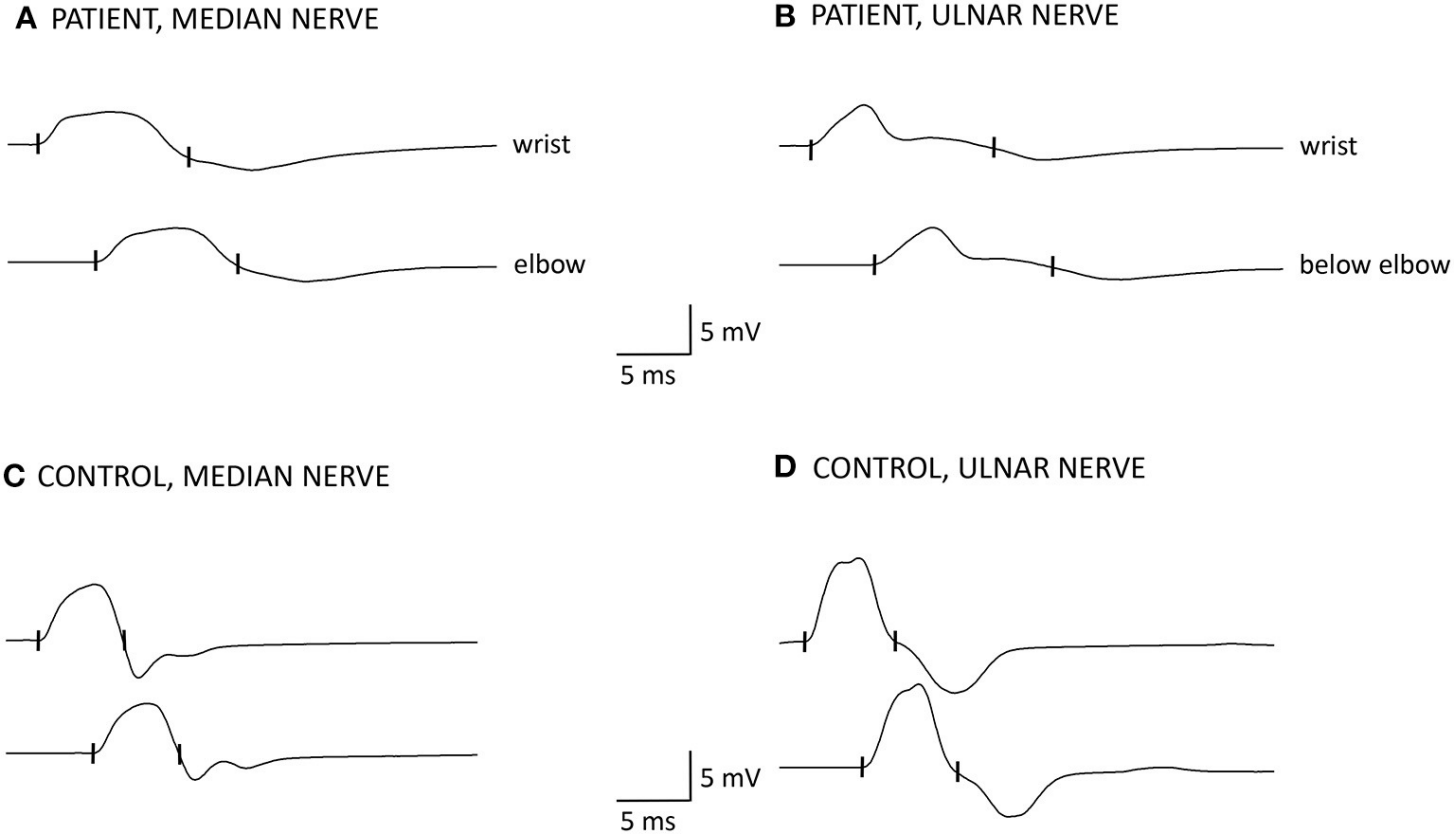
Motor nerve conduction studies of patient 1 **(A,B)** compared to a healthy control subject **(C,D)**. Amplitude and duration of the negative phase of compound muscle action potentials (CMAPs) were measured at a sensitivity of 0.5 mV with a 2 Hz low frequency filter. The cut-off values for distal CMAP duration are according to reported normal values + 2 SD (12). **(A)** median nerve: distal CMAP amplitude is reduced (4.2 mV), distal motor latency (DML) is normal (3.9 ms), distal CMAP duration is increased (9.3 ms, 127% of upper limit of normal = 7.3 ms), conduction velocity (CV) is 48 m/s. CMAP amplitude and duration did not change between proximal and distal stimulation. Note the broadening and smooth contour of the negative phase of the distal CMAP and the reduction of the ensuing positive phase compared to panel **(C)** (CMAP duration = 5.7 ms); **(B)** ulnar nerve: distal CMAP amplitude is slightly reduced (5.7 mV), DML is normal (2.9 ms), distal CMAP duration is increased (13.7 ms, 183% upper limit of normal = 7.5 ms), CV is 55 m/s. CMAP amplitude and duration did not change with proximal stimulation. Note the very prolonged negative phase of the distal CMAP with a long tail and the absence of the ensuing positive phase compared to panel **(D)** (CMAP duration = 6.3 ms).

Motor nerve conduction studies showed normal distal latencies and normal conduction velocities. Distal compound muscle action potential (CMAP) amplitudes were decreased and CMAP durations were prolonged in median and ulnar nerves in both patients (Table 2, Figure 2). Sensory conduction velocities and sensory nerve action potential amplitudes were normal. Needle EMG showed spontaneous activity (fibrillation potentials) in patient 2 and a myopathic pattern with short duration motor unit action potentials, increased percentage of polyphasic potentials, and early recruitment at voluntary effort in proximal muscles in both patients. Distal muscles were unremarkable. Within 2 weeks from admission in neurorehabilitation, serum CK returned to normal values (23 and 201 U/l, respectively).

Clinical condition improved progressively in both patients, who were discharged home after 6–8 weeks of rehabilitation, with a muscle strength of 3/5 in proximal upper limb and 4/5 in proximal lower limb muscles, and normal walking capability. However, both complained of reduced endurance and increased fatigue during physical activity.

## Discussion

In the presented patients, clinical, laboratory and electrodiagnostic findings were consistent with a myopathy except for increased distal CMAP duration that is usually considered a hallmark of acquired demyelination. However, prolonged duration of distal CMAPs that did not change between distal and proximal stimulation (Figure 2), together with normal distal motor latencies and conduction velocities, indicates that in these patients, temporal dispersion of distal CMAP is due to slow muscle fibers conduction velocity. Indeed, prolonged distal CMAP duration, besides reduced CMAP amplitude, has previously been reported in patients with CIM, who presented, as compared to healthy controls, with reduced mean muscle fiber conduction velocity, which was inversely related to CMAP duration (13, 14). Moreover, in an *in vitro* model, sera from patients with CIM applied to single muscle fibers induced depolarization of the resting membrane potential, reduced the action potential rise time, and increased inward sodium current peak amplitude (15). Evidence from human studies and animal models indicates that in CIM associated with sepsis (the so-called “SIM,” sepsis-induced myopathy), systemic inflammatory response, and cytokine release induce a depolarizing shift of the muscle cell membrane potential, sodium channel inactivation, slowing of muscle fiber conduction velocity until total membrane inexcitability, increase of membrane permeability for Ca^2+^, and eventually Ca^2+^-dependent muscle necrosis by proteasome activation (16).

We hypothesize that in the reported patients, by analogy with SIM, myopathy was caused by the COVID-associated hyper-inflammatory state, as demonstrated by high initial serum levels of CRP and IL-6. Prolonged distal CMAP durations can be explained by muscle membrane hypo-excitability combined with slow muscle fiber conduction velocity in regenerating muscle fibers.

Interestingly also six reported COVID-10 patients with acute quadriplegic myopathy (8), showed markedly prolonged CMAP durations without evidence of acute myonecrosis (CK were slightly elevated in half patients and decreased in few days) and, with exception of one patient who died due to sepsis, showed rapid improvement of weakness (14–20 days). This can concur with the proposed mechanism of muscular impairment in COVID-19, ranging from membrane excitability dysfunction (which reflects in reduced amplitude and increased duration of CMAPs) with possible prompt recovery to myonecrosis, CK elevation, consequent muscle atrophy, and poorer outcome.

Serum IL-6 elevation is common in critically ill patients (16), but it also plays a central role in the COVID-19 inflammation cascade already at an early stage, preceding need for intensive care, and it correlates with disease severity (17).

In conclusion, the same pathogenetic mechanism that causes interstitial pneumonia and damage to extrapulmonary tissues and organs in COVID-19, i.e., the inflammatory cytokine storm together with coagulopathy and macrophage activation, could contribute, in patients requiring prolonged critical care, to skeletal muscle damage (17).

Further studies are necessary to elucidate the pathogenesis of COVID-19-associated myopathy and to differentiate among direct infection, autoimmune process, and CIM due to hyperinflammation; in particular, muscle biopsy with specific investigations would be of crucial importance.

## Data Availability Statement

The original contributions presented in the study are included in the article/supplementary material, further inquiries can be directed to the corresponding author/s.

## Ethics Statement

Ethical review and approval was not required for the study on human participants in accordance with the local legislation and institutional requirements. Written informed consent for participation was not required for this study in accordance with the national legislation and the institutional requirements. Written informed consent was obtained from the patients for the submission of their data and for the publication of any potentially identifiable images or data included in this article.

## Author Contributions

Material preparation, data collection, and analysis were performed by VV, LSe, and DF. The first draft of the manuscript was written by VV and MK. AU contributed substantially to the interpretation of the results, provided critical feedback, and revised the manuscript. All authors contributed in review and editing of the manuscript and approved its final version. All authors contributed to the study conception and design.

## Conflict of Interest

The authors declare that the research was conducted in the absence of any commercial or financial relationships that could be construed as a potential conflict of interest.
